# Vitamin D alleviates lead induced renal and testicular injuries by immunomodulatory and antioxidant mechanisms in rats

**DOI:** 10.1038/s41598-018-23258-w

**Published:** 2018-03-19

**Authors:** Mohammad A. BaSalamah, Abdelghany Hassan Abdelghany, Mohamed El-Boshy, Jawwad Ahmad, Shakir Idris, Bassem Refaat

**Affiliations:** 1 Pathology Department, Faculty of Medicine, Umm Al-Qura University, Al Abdeyah, Makkah, Saudi Arabia; 2 Department of Anatomy, Faculty of Medicine, Alexandria University, Alexandria, Egypt; 3 Laboratory Medicine Department, Faculty of Applied Medical Sciences, Umm Al-Qura University, Al Abdeyah, PO Box, 7607 Makkah, Saudi Arabia; 4 Department of Clinical Pathology, Faculty of Veterinary Medicine, Mansoura University, Mansoura, Egypt

## Abstract

This study measured the effects of vitamin D (VD) supplementation on the underlying molecular pathways involved in renal and testicular damage induced by lead (Pb) toxicity. Thirty two adult male Wistar rats were divided equally into four groups that were treated individually or simultaneously, except the negative control, for four weeks with lead acetate in drinking water (1,000 mg/L) and/or intramuscular VD (1,000 IU/kg; 3 days/week). Pb toxicity markedly reduced serum VD and Ca^2+^, induced substantial renal and testicular injuries with concomitant significant alterations in the expression of VD metabolising enzymes, its receptor and binding protein, and the calcium sensing receptor. Pb also significantly promoted lipid peroxidation and pro-inflammatory cytokines (IL-4 and TNF-α) in the organs of interest concomitantly with declines in several anti-oxidative markers (glutathione, glutathione peroxidase and catalase) and the anti-inflammatory cytokine, IL-10. The co-administration of VD with Pb markedly mitigated renal and testicular injuries compared with positive controls. This was associated with restoration of the expression of VD related molecules, promotion of anti-oxidative and anti-inflammatory markers, but tissue Pb concentrations were unaffected. In conclusion, this report is the first to reveal potential protective effects for VD against Pb-induced renal and testicular injuries via anti-inflammatory and anti-oxidative mechanisms.

## Introduction

Lead (Pb) is a non-essential element that could result in serious health problems due to toxicity arising from environmental pollution^1–3^. The risk is significant with the World Health Organisation (WHO) reporting 853,000 deaths related to Pb toxicity in 2013^4^. Ingesting contaminated food and water is the route of lead intoxication for the general population, while inhalation of polluted dust and fumes is more common in the occupational setting^2^. Accumulation of Pb in tissues induces cellular damage through oxidative stress following overproduction of reactive oxygen species (ROS) and reduction in the activities of cellular antioxidant system^5–8^. The metal also inhibits cellular energy production and induces apoptosis subsequent to mitochondrial impairment and DNA damage^9,10^. Additionally, Pb simultaneously upregulates and inhibits the production of several pro- and anti-inflammatory cytokines^11,12^.

Pb poisoning provokes severe multiorgan damage, with chronic exposure increasing the risk of developing renal diseases^1–3^, and adverse reproductive consequences^5,13^. In this context, blood Pb levels greater than 60 μg/dL were shown to induce nephropathies characterised by tubular dysfunction and decreased creatinine clearance^2,3,14^. Prolonged exposure to Pb has also been reported to cause abnormal sex hormones levels and significantly lower sperm count that were morphologically abnormal and immotile^15,16^. The standard clinical management of lead poisoning encompasses the administration of chelators (e.g. succimer) and ensuring the avoidance of further exposure to contaminated sources^4,17^. However, controversies still surround the efficacy of chelators, since they are mainly capable of eliminating the metal from circulation, with little effects on tissue precipitates^18–20^. Therefore, there is still a compelling need to develop more potent chelators that could efficiently protect against Pb-induced tissue damage^18–20^.

Vitamin D (VD) is a steroid hormone that is mainly synthesised as a prohormone in the skin following exposure to sunlight and the production of active VD (VD_3_) occurs in renal proximal tubular cells by the actions of 1α hydroxylase (Cyp27b1) enzyme^21,22^. The transportation of VD in circulation is achieved by its binding protein (VDBP) and the hormone activities are mainly controlled by its catabolising enzyme, Cyp24a1^21^. VD receptor (VDR) is located in the cytoplasm, and once activated, the receptor forms a complex with other nuclear receptors known as retinoid X receptors (RXR)^23^. The VDR/RXR complex then interacts with VD responsive elements on target genes to control their expression^23^. VD has a wide range of cytoprotective actions that include anti-fibrotic, anti-oxidative and anti-inflammatory effects, in addition to its known skeletal effects^22,24^. The classical actions of VD on Ca^2+^ homeostasis involve the regulation of several cellular proteins including the cell membrane calcium sensing receptor (CaSR)^25^. The activation of this receptor, which is coupled with G-protein, regulates the balance between intra- and extracellular Ca^2+^^25^. CaSR has been localised in several tissues, including kidney and testis, and its cellular expression has been shown to be regulated by VD^26^.

Little is currently known about the links between VD and Pb toxicity, and the available data is controversial. While polymorphisms in VDR gene have been associated with increases in blood Pb levels^27,28^, others have also reported significant negative correlations between blood levels of Pb and VD^29,30^. Additionally, numerous studies suggested that Pb enters the cytoplasm through Ca^2+^ channels^31,32^ and that Ca^2+^ channel blockers resulted in lower levels of Pb in renal tissues^31^. In contrast, Ca^2+^ consumption above the Dietary Reference Intake was associated with lower blood Pb levels^30^. The present study, was therefore designed to measure the effects VD_3_ supplementation on renal and testicular damage during chronic lead intoxication in rats together with the expression profiles of VD related molecules, oxidative stress markers and a panel of pro- and anti-inflammatory cytokines in the tissues of interest.

## Results

### Lead concentrations in renal and testicular tissues

The concentrations of Pb in renal tissues were markedly elevated in the positive control (PC) group (8.05 ± 3.12 µg/g) and the group treated simultaneously with both Pb and VD_3_ (P-VD) (7.12 ± 2.98 µg/g) compared with the negative control group (NC) (0.124 ± 0.04 µg/g; P = 0.02 × 10^−16^ and P = 0.03 × 10^−11^, respectively) and the normal animals treated with VD_3_ alone (N-VD) (0.094 ± 0.03 µg/g; P = 0.01 × 10^−17^ and P = 0.02 × 10^−13^, respectively). Similarly, testicular specimens form the PC (5.45 ± 2.32 µg/g) and P-VD (4.92 ± 2.18 µg/g) groups had significantly higher concentrations of lead than the NC (0.114 ± 0.03 µg/g; P = 0.04 × 10^−8^ and P = 0.05 × 10^−6^, respectively) and N-VD (0.088 ± 0.04 µg/g; P = 0.02 × 10^−10^ and P = 0.03 × 10^−7^, respectively) groups. However, there were no significant differences (P > 0.05) between the PC and P-VD groups in renal and testicular Pb concentrations.

### Biochemical Findings

The N-VD group had significantly higher levels of serum 25-OH VD (28.5 ± 5.20 ng/mL; P = 0.03) compared with the NC (23.05 ± 3.08 ng/mL). However, the serum concentrations of urea (38.66 ± 3.8 vs. 37.16 ± 3.6 mg/dL), creatinine (0.49 ± 0.04 vs. 0.55 ± 0.1 mg/dL), calcium (10.30 ± 0.38 vs. 9.90 ± 0.64 mg/dL) and total testosterone (4.40 ± 1.44 vs. 3.95 ± 0.91 ng/dL) were comparable between the N-VD and NC groups. The PC group showed markedly elevated serum creatinine (P = 0.0001) and urea (P = 0.00006), together with significantly lower levels of serum 25-OH vitamin D (P = 0.01), calcium (P = 0.005) and total testosterone (P = 0.009), compared with the NC group (Table 1). The simultaneous administration of VD_3_ with Pb significantly alleviated the effects of the heavy metal on serum creatinine (P = 0.007), urea (P = 0.03), 25-OH VD (P = 0.01), Ca^2+^ (P = 0.02) and total testosterone (P = 0.009) compared with the PC group. Additionally, the serum levels of all biochemical parameters were comparable between the P-VD and NC groups, except for urea, which was significantly higher in the former group (Table 1).

**Table 1:** Mean ± SD of serum renal function parameters, calcium, 25-OH VD and total testosterone in the different study groups.

Serum Parameters	Study Groups		
	NC (n = 8)	PC (n = 8)	P-VD (n = 8)
Urea (mg/dL)	37.16 ± 3.6	55.83 ± 5.7^b^	45.80 ± 4.1^b,c^
Creatinine (mg/dL)	0.55 ± 0.1	0.78 ± 0.09^b^	0.59 ± 0.07^d^
Calcium (mg/dL)	9.90 ± 0.64	8.63 ± 0.58^a^	9.72 ± 0.48^c^
25-OH VD (ng/mL)	23.05 ± 3.08	15.33 ± 4.13^b^	20.16 ± 2.04^c^
Testosterone (ng/mL)	3.95 ± 0.91	2.38 ± 0.48^b^	3.60 ± 0.61^d^

(^a^P < 0.05 compared with the NC group; ^b^P < 0.01 compared with the NC group; ^c^P < 0.05 compared with the PC group and ^d^P < 0.01 compared with the PC group).

### Serum concentrations of targeted cytokines

Lead poisoning induced significant increases in serum IL-4 (P = 0.0008) and TNF-α (P = 0.001) that coincided with a significant decrease in serum IL-10 concentrations (P = 0.0003) compared with the NC group (Table 2). TNF-α and IL-10 were restored in the P-VD group to the levels observed in the NC group, and both cytokines were significantly different compared with the PC animals (P = 0.003 and P = 0.005, respectively). Although the serum concentrations of IL-4 were also significantly decreased in the P-VD compared with the PC group (P = 0.02), they remained significantly higher in this group (P = 0.0004) than the NC group (Table 2). The administration of VD_3_ in normal rats did not alter the levels of IL-4 (7.51 ± 1.04 pg/mL), TNF-α (23.66 ± 3.62 pg/mL) and IL-10 (11.51 ± 1.51 pg/mL) compared with the NC rats (P > 0.05).

**Table 2:** Mean ± SD of serum TNF-α, IL-4 and IL-10 in the different study groups.

Serum cytokines (pg/mL)	Study Groups		
	NC (n = 8)	PC (n = 8)	P-VD (n = 8)
TNF-α	25.01 ± 3.73	37.33 ± 7.14^b^	27.51 ± 4.81^c^
IL-4	7.83 ± 1.60	15.66 ± 2.33^b^	11.16 ± 2.04^b,c^
IL-10	12.66 ± 2.22	7.01 ± 1.78^b^	10.33 ± 1.86^d^

(^a^P < 0.05 compared with the NC group; ^b^P < 0.01 compared with the NC group; ^c^P < 0.05 compared with the PC group and ^d^P < 0.01 compared with the PC group).

### Renal and testicular lipid peroxidation and antioxidant markers

There was no significant difference in the levels of malondialdehyde (MDA), catalase (CAT) and superoxide dismutase (SOD) in renal and testicular tissues between the NC and N-VD groups. However, The administrations of VD_3_ to normal animals induced significant elevations in renal and testicular levels of glutathione (GSH) (12.9%; P = 0.03 and 21.4%; P = 0.02, respectively) and glutathione peroxidase (GPx) (44.5% ± 4.4; P = 0.009 and 72.9% ± 11.9; P = 0.0005, respectively) compared with the control group.

Prolonged exposure to Pb, within the PC group compared to the NC group, resulted in significant increases in renal (P = 0.0001) and testicular (P = 0.00005) tissues levels of MDA by 36% and 53%, respectively (Table 3). The levels of MDA in the kidney (P = 0.001) and testis (P = 0.03), obtained from the P-VD group and compared with PC, were significantly reduced by 22.8% and 16%, respectively. Although renal MDA concentrations were similar between the P-VD and NC groups, the lipid peroxidation marker remained significantly higher (P = 0.01) in the P-VD testicular tissues (Table 3).

**Table 3:** Mean ± SD of lipid peroxidation and antioxidant markers in renal and testicular tissue homogenates from all study groups.

Tissue Parameters		Study Groups		
		NC (n = 8)	PC (n = 8)	P-VD (n = 8)
MDA (nmol/g)	*Kidney*	33.16 ± 4.75	45.10 ± 4.97^b^	34.81 ± 3.85^d^
	*Testis*	25.16 ± 3.97	38.66 ± 5.61^b^	32.50 ± 3.10^b,c^
GSH (mg/g)	*Kidney*	38.82 ± 5.31	29.65 ± 3.32^b^	36.34 ± 4.54^d^
	*Testis*	24.17 ± 3.37	19.02 ± 3.78^b^	22.16 ± 3.31
GPx (µg/mg)	*Kidney*	3.93 ± 0.95	1.82 ± 0.70^b^	3.52 ± 0.41^d^
	*Testis*	2.63 ± 0.98	1.58 ± 0.30^a^	2.40 ± 0.48^c^
CAT (U/mg)	*Kidney*	245.8 ± 34.9	175.1 ± 17.8^b^	222.6 ± 15.4^d^
	*Testis*	95.33 ± 16.9	65.50 ± 14.8^a^	92.30 ± 9.8^d^
SOD (U/mg)	*Kidney*	103.2 ± 9.8	97.66 ± 16.2	104.51 ± 10.11
	*Testis*	12.1 ± 2.28	10.66 ± 1.96	11.33 ± 2.71

(^a^P < 0.05 compared with the NC group; ^b^P < 0.01 compared with the NC group; ^c^P < 0.05 compared with the PC group and ^d^P < 0.01 compared with the PC group).

In contrast, the PC group showed prominent reductions in renal levels of GSH (23.6%; P = 0.002), GPx (53.7%; P = 0.001) and CAT (28.7%; P = 0.0009) than the NC group. Similarly, testicular levels of GSH (P = 0.04), GPx (P = 0.01) and CAT (P = 0.03) were also significantly decreased by 22.3%, 40% and 31.2%, respectively, in the PC group compared with the NC rats. However, there was no significant difference in the levels of SOD in both tissues between both groups (Table 3). The simultaneous administration of VD_3_ with Pb rescued the observed decreases in renal and testicular antioxidant markers, and the levels were then comparable to the NC group. All markers were significantly higher than the PC group, except for testicular GSH, which showed a non-significant decrease (Table 3).

### Effects of vitamin D_3_ on lead induced renal and testicular cell damage

**Figure 1:**
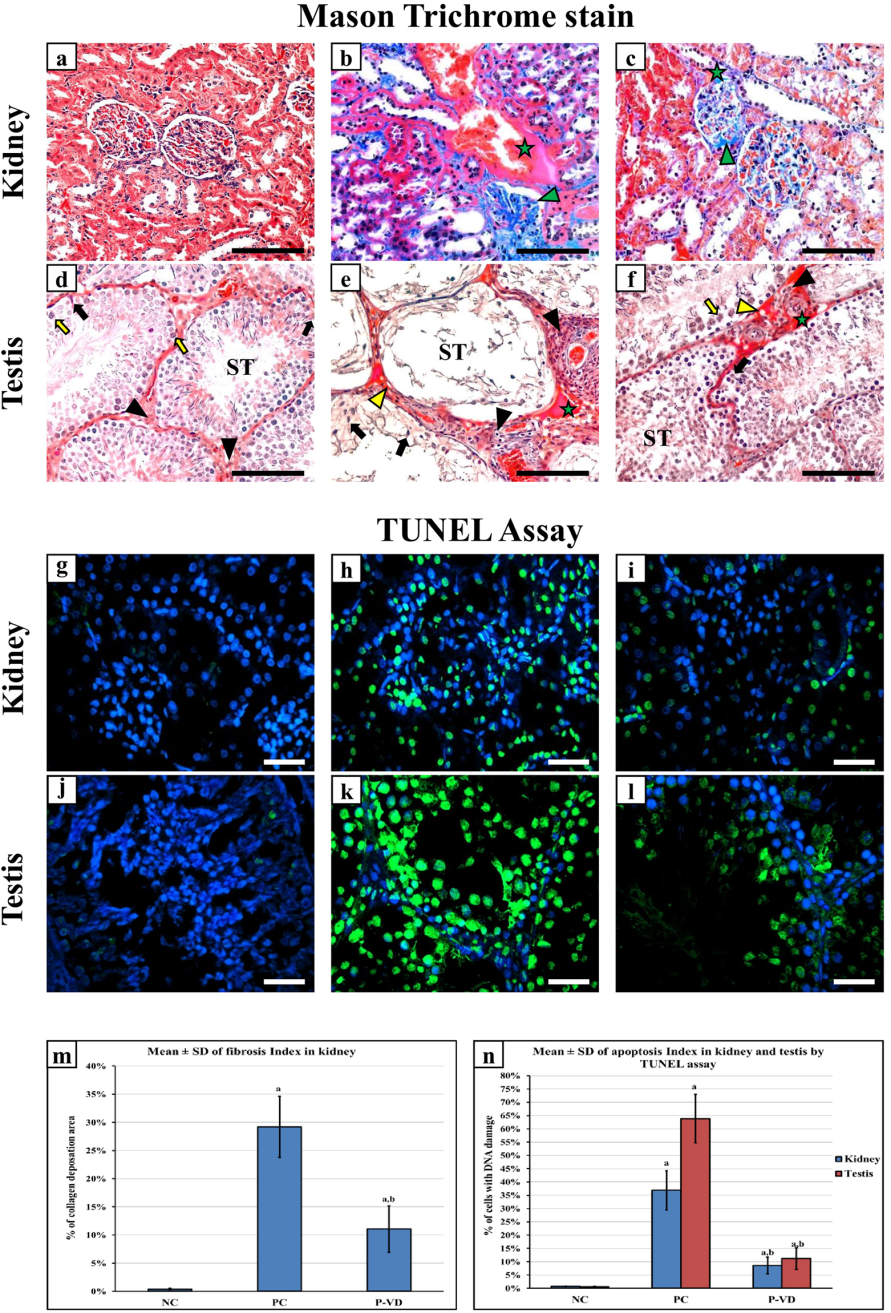
Histopathological features by Mason’s trichrome stain of renal and testicular tissues of the NC (**a** & **d**), PC (**b** & **e**), and P-VD (**c** & **f**) groups (40× objective, scale bar = 8 µm; green star = interstitial necrosis; green arrow head = collagen type I deposition in glomerulus; yellow arrow head = hyaline droplets; ST = seminiferous tubule; black arrow = Sertoli cell; black arrow head = Leydig cell and yellow arrow = spermatogonia). Cellular DNA damage was detected by TUNEL assay (Green fluorescent dye) in kidney and testis from the NC (**a** & **g**), PC (**b** & **h**), and P-VD (**c** & **i**) groups (40× objective; scale bar = 10 µm; positive nuclei are stained in green). Additionally, the renal fibrosis index (m) and renal and testicular apoptosis/necrosis index (n) are shown as bar graphs for the study groups. (a = P < 0.05 compared with NC group and b = P < 0.05 compared with PC group).

Renal (Fig. 1a) and testicular (Fig. 1d) tissues from the NC group showed normal architecture with minimal/negligible collagen type I deposition by Mason trichrome stain. Coherently, a handful scattered apoptotic bodies were observed in normal kidney (Fig. 1g) and testis (Fig. 1j) using the TUNEL assay. Pb intoxication induced interstitial necrosis in kidneys and testicles of the PC group and was observed to be more pronounced in the testicular specimens. In more detail, renal tissue damage showed several areas of interstitial necrosis, glomerular and inter-tubular fibrosis, widening of tubular lumens and protrusion of tubular cell nuclei (Fig. 1b) with a significant increase in cell DNA damage (Fig. 1h). Pb-induced testicular damage showed severe interstitial hyaline degeneration, seminiferous tubule atrophy with almost complete loss of both germ and Sertoli cells (Fig. 1e), and many cells were also positive for apoptotic bodies (Fig. 1k).

VD_3_ significantly decreased the amount of renal fibrosis (Fig. 1c) and the numbers of apoptotic bodies (Fig. 1i) in the P-VD group. Similar effects were also seen in testicular specimens, and the degeneration of interstitial tissue was significantly decreased with preservation of seminiferous tubule morphology, salvation of Sertoli and germ cells (Fig. 1f) and a significantly lower number of apoptotic bodies (Fig. 1l). Renal fibrosis index (Fig. 1m) and apoptosis/necrosis index of renal and testicular tissues (Fig. 1n) from all groups are shown as graph bars.

### Immunohistochemistry of vitamin D related proteins and targeted cytokines

**Figure 2:**
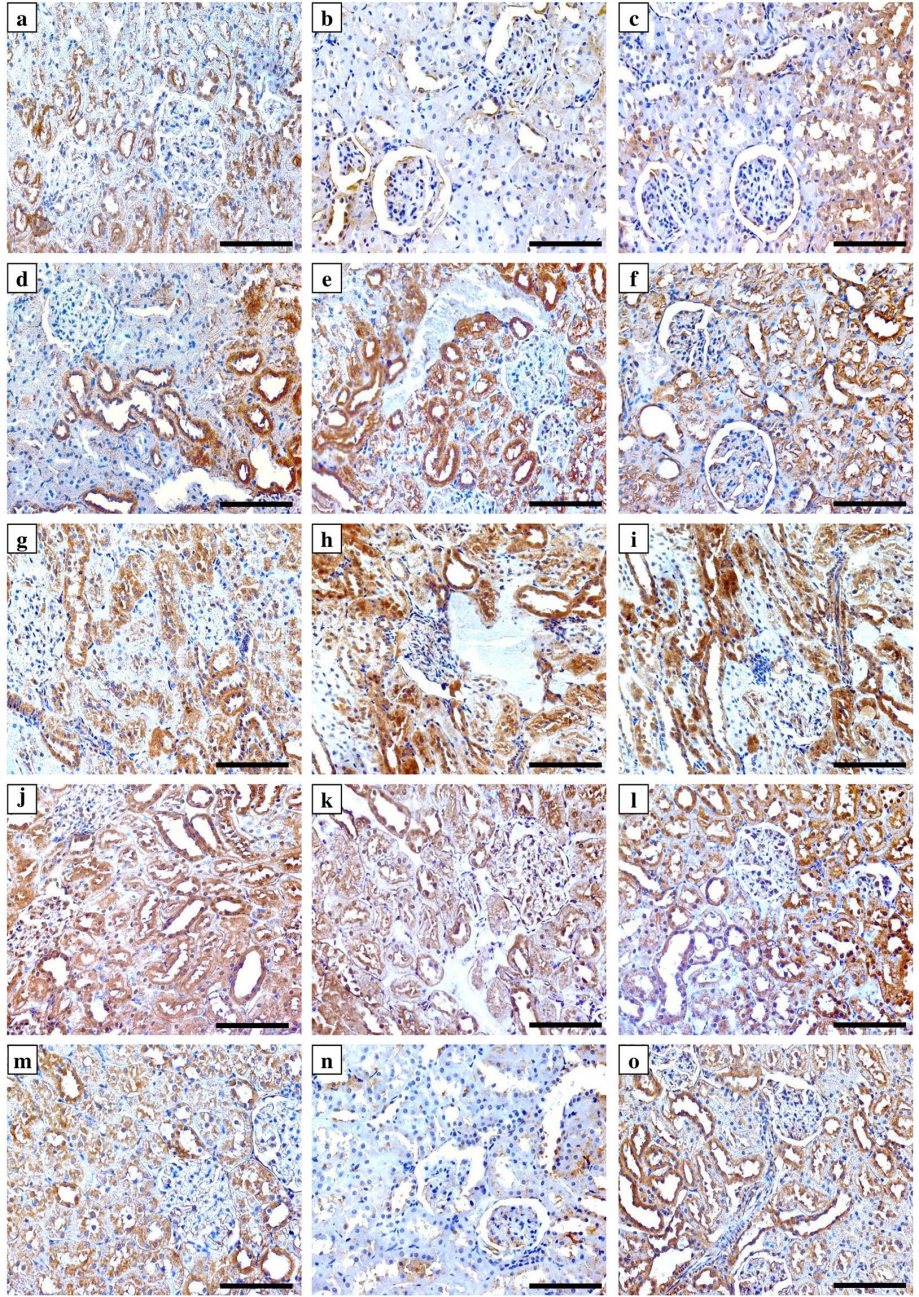
Immunohistochemical expression of Cyp27b1 enzyme (upper row), Cyp24a1 enzyme (2^nd^ row from top), VDBP (3^rd^ row from top), VDR (4^th^ row from top) and CaSR (bottom row) in kidney sections from the NC (left column), PC (middle column) and P-VD (right column) groups (40× objective, scale bar = 8 µm).

**Figure 3:**
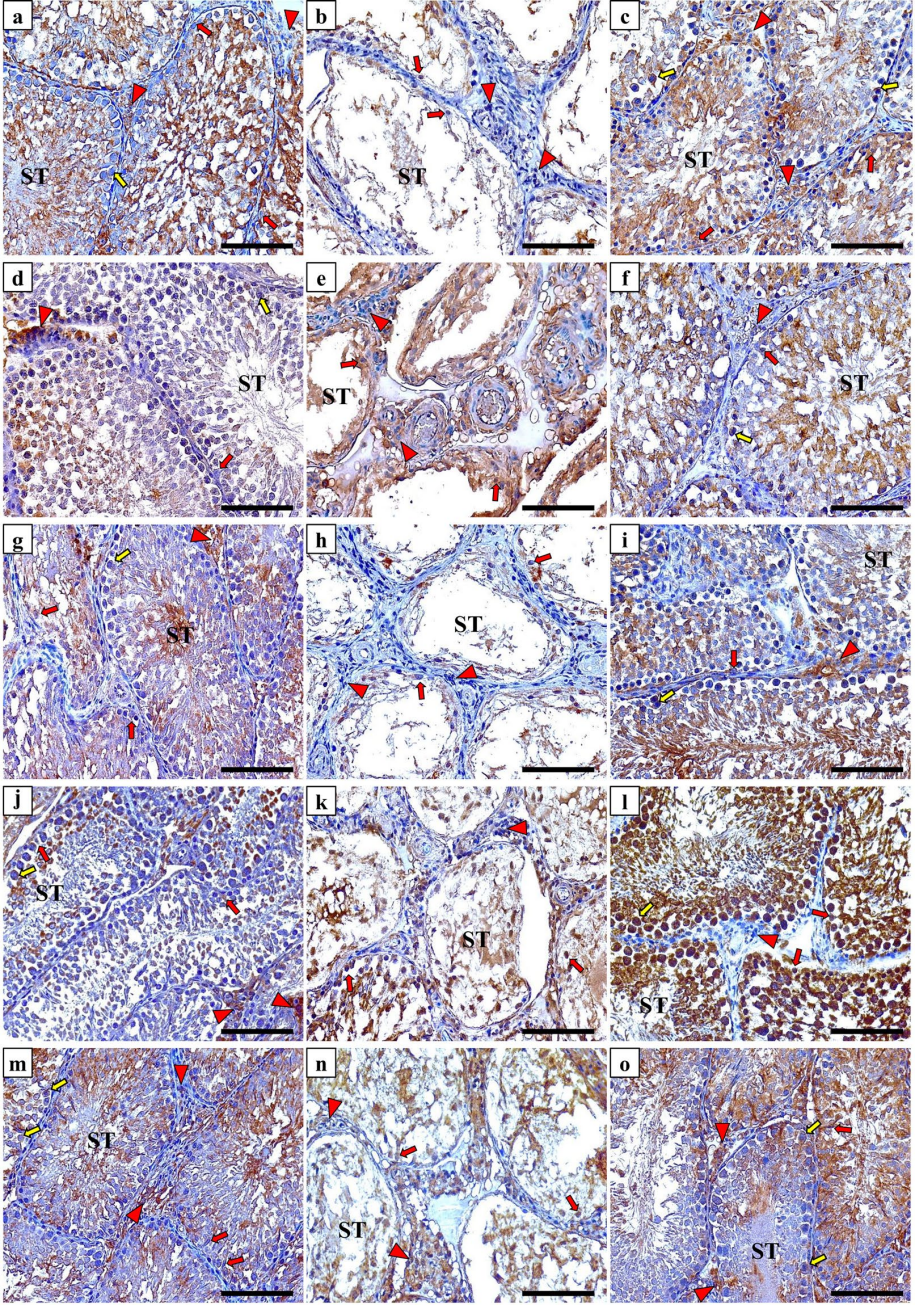
Immunohistochemical expression of Cyp27b1 enzyme (upper row), Cyp24a1 enzyme (2^nd^ row from top), VDBP (3^rd^ row from top), VDR (4^th^ row from top) and CaSR (bottom row) in testicular sections from the NC (left column), PC (middle column) and P-VD (right column) groups (40× objective; scale bar = 8 µm; ST = seminiferous tubule; red arrow = Sertoli cell; red arrow head = Leydig cell and yellow arrow = spermatogonia).

**Figure 4:**
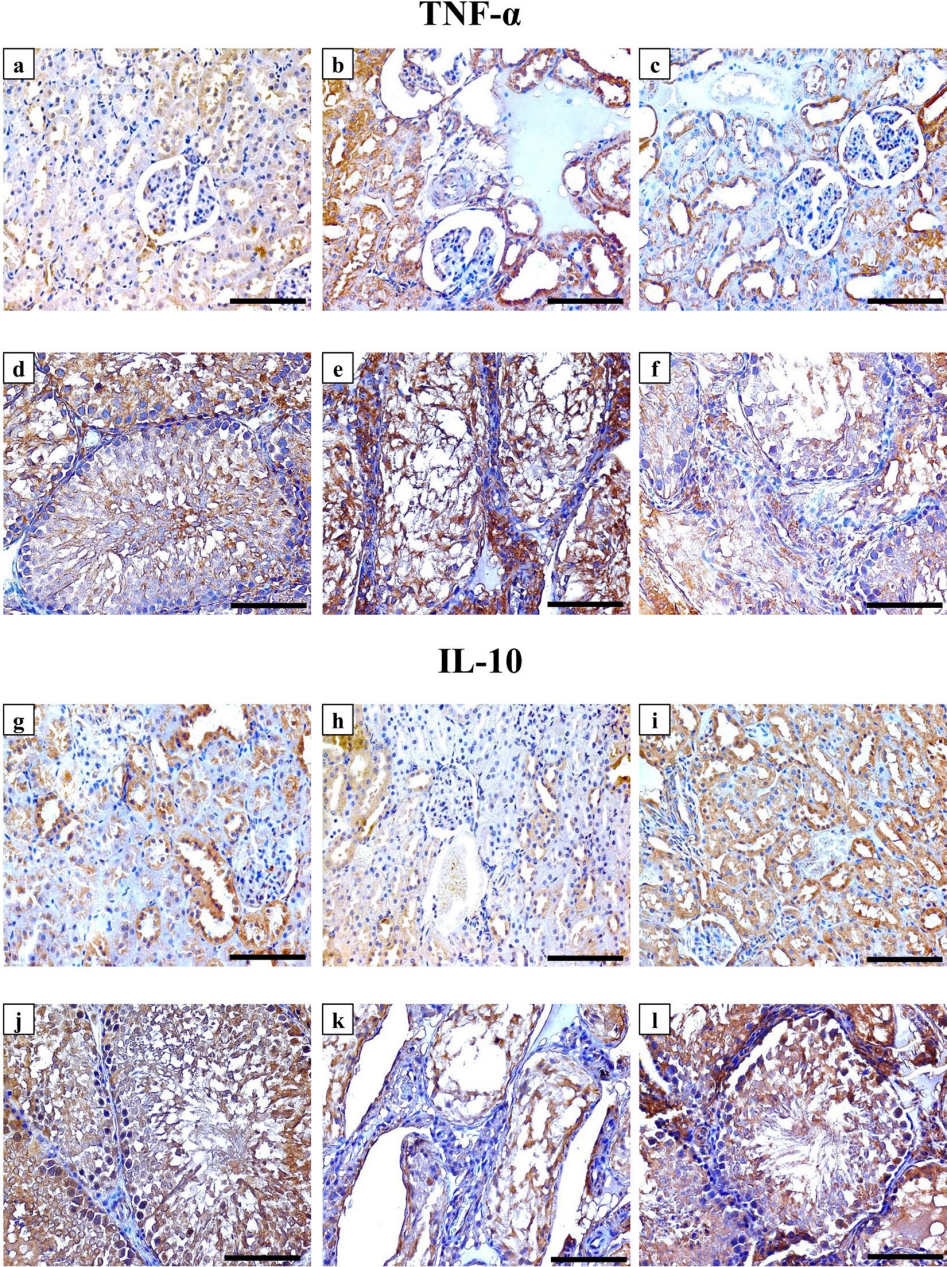
Immunohistochemical expression of TNF-α and IL-10 in renal (1^st^ and 3^rd^ rows from top) and testicular (2^nd^ and 4^th^ rows from top) sections from the NC (left column), PC (middle column) and P-VD (right column) groups (40× objective, scale bar = 8 µm).

All target molecules were detected and the expression was localised in the renal and testicular tissues of the NC group. The antibodies against Cyp27b1 (Fig. 2a), Cyp24a1 (Fig. 2d), VDBP (Fig. 2g) and CaSR (Fig. 2m) clearly labelled the cytoplasm and apical border of renal tubular cells of the NC animal tissues, except for VDR, which showed nuclear localisation in glomerular and tubular cells (Fig. 2j). All VD related proteins (Fig. 3; left column) were also expressed by Sertoli cells and spermatogonium, in addition to primary and secondary spermatocytes within the seminiferous tubules of the NC group tissues. Furthermore, positive immunostain for Cyp27b1 (Fig. 3a), VDR (Fig. 3j) and CaSR (Fig. 3m) was also observed in Leydig cells. The expression of TNF-α and IL-10 was also detected in renal (Fig. 4, panels a & g) and testicular (Fig. 4, panels d & j) specimens of the NC group.

The PC group showed significant decreases than the NC group in the renal tissue expression of Cyp27b1 enzyme (Fig. 2b), VDR (Fig. 2k), CaSR (Fig. 2n) and IL-10 (Fig. 4b), especially around the damaged areas. Furthermore, there was a significant increase in renal Cyp24a1 (Fig. 2e), VDBP (Fig. 2h) and TNF-α (Fig. 4b) compared with the NC group (Table 4). Moreover, Pb poisoning also induced similar effects in testicular tissues in relation to the expression of VD synthesising (Fig. 3b) and catalysing (Fig. 3e) enzymes, VDR (Fig. 3k), CaSR (Fig. 3n), TNF-α (Fig. 4e) and IL-10 (Fig. 4h) compared with controls. However, there was a significant decrease in the expression of testicular VDBP (Fig. 3h) in the PC group (Table 4).

**Table 4:** Mean ± SD of immunohistochemistry scores for Cyp27b1, Cyp24a1, VDBP, VDR, CaSR, TNF-α and IL-10 in renal and testicular specimens from the different study groups.

Immunohistochemistry arbitrary scores		Study Groups		
		NC (n = 8)	PC (n = 8)	P-VD (n = 8)
Cyp27b1	*Kidney*	148.2 ± 28.7	100.7 ± 31.1^a^	174.6 ± 30.1^c^
	*Testis*	423.8.2 ± 52.6	89.3 ± 24.8^b^	516.3.6 ± 52.3^a,c^
Cyp24a1	*Kidney*	167.3 ± 34.4	354.5 ± 39.2^b^	292.3 ± 27.2^a,c^
	*Testis*	108.4 ± 23.9	169.2 ± 33.3^a^	365.7 ± 34.9^a,c^
VDBP	*Kidney*	319.7 ± 28.5	363.7 ± 37.6^a^	314.2 ± 37.3^b^
	*Testis*	319.8 ± 46.4	159.3 ± 28.9^b^	448.8 ± 51.7^a.c^
VDR	*Kidney*	471.6 ± 40.1	277.6 ± 23.1^b^	394.7 ± 22.6^a,d^
	*Testis*	501.7 ± 57.4	350.1 ± 51.6^a^	732.4 ± 61.1^a,d^
CaSR	*Kidney*	202.8 ± 29.4	62.8 ± 24.2^b^	264.2 ± 29.6^a,d^
	*Testis*	479.8 ± 43.9	334.6 ± 22.2^a^	462.8 ± 34.1^c^
TNF-α	*Kidney*	88.1 ± 19.2	215.9 ± 33.6^b^	152.7 ± 24.6^a,c^
	*Testis*	243.6 ± 25.1	455.6 ± 37.7^b^	173.5 ± 21.9^a,c^
IL-10	*Kidney*	153.9 ± 26.9	76.8 ± 27.1^a^	241.2 ± 22.9^b,d^
	*Testis*	527.2 ± 42.3	168.3 ± 19.8^a^	510.4 ± 30.2^d^

(^a^P < 0.05 compared with the NC group; ^b^P < 0.01 compared with the NC group; ^c^P < 0.05 compared with the PC group and ^d^P < 0.01 compared with the PC group).

The abnormal expression of VD associated molecules were restored in the P-VD group renal (Fig. 2; right column) and testicular (Fig. 3; right column) tissues compared with the PC group and the scores were comparable to control tissues (Table 4). Additionally, VD_3_ significantly downregulated the expression of TNF-α (Fig. 4; panels c & f) and promoted the expression of IL-10 (Fig. 4; panels i & l) in the kidney and testis (Table 4).

### Renal and testicular gene expression of targeted molecules

**Figure 5:**
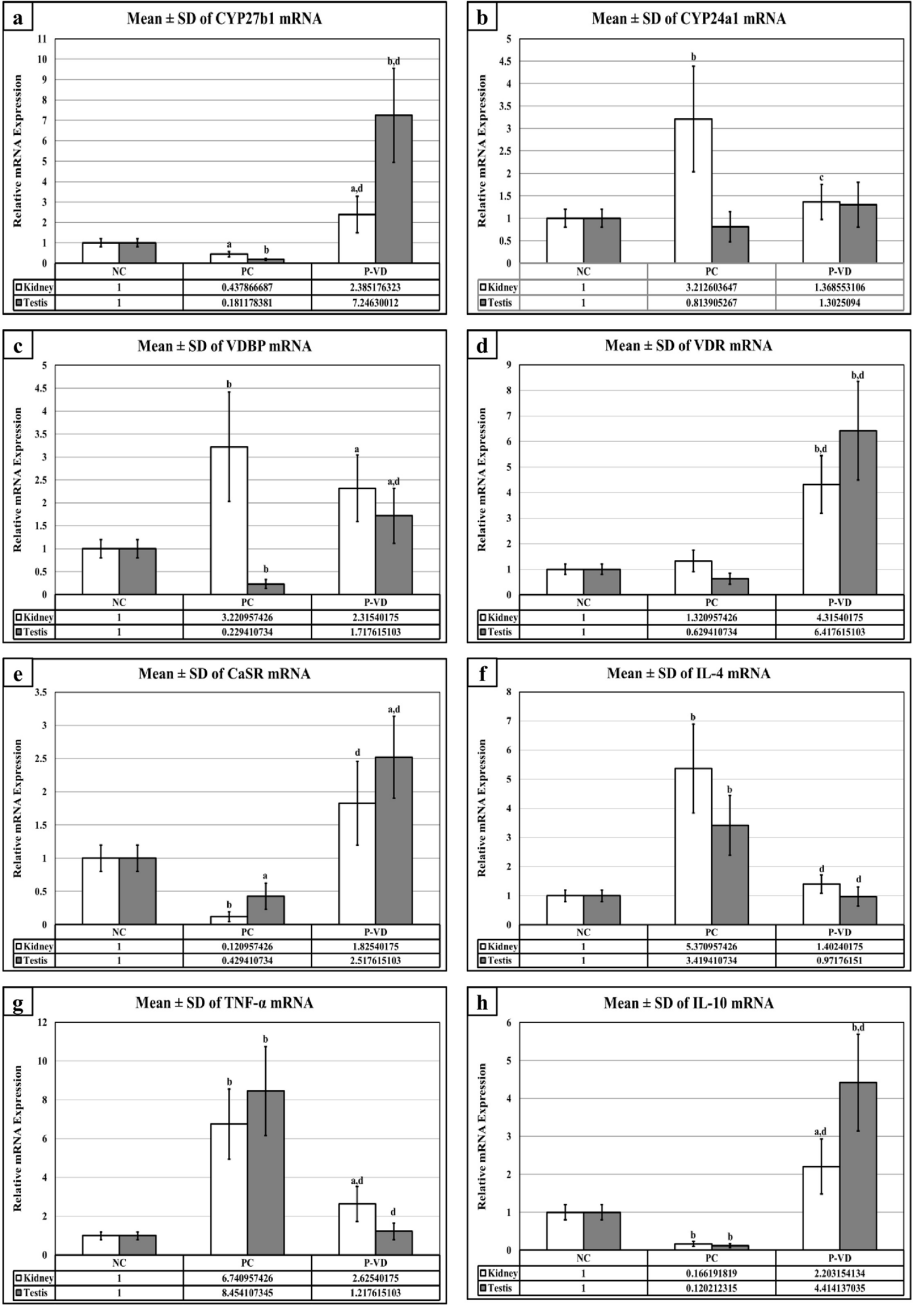
Mean ± SD of messenger RNA relative expression of (**a**) *Cyp27b1*, (**b**) *Cyp24a1*, (**c**) *VDBP*, (**d**) *VDR*, (**e**) *CaSR*, (**f**) *IL-4*, (**g**) *TNF-α* and (**h**) *IL-10* in the different study groups. (a = P < 0.05 compared with the NC group; b = P < 0.01 compared with the NC group; c = P < 0.05 compared with the PC group and d = P < 0.01 compared with the PC group).

In agreement with the immunohistochemistry results, the gene expression experiments revealed a significant decrease in the mRNAs of *Cyp27b1* (2 folds), *CaSR* (10 folds) *and IL-10* (10 folds) in kidney specimens from the PC group compared with NC. Additionally, there was a significant increase in the renal gene expression of *Cyp24a1* (3 folds), *VDBP* (3 folds), *IL-4* (5 folds) and *TNF-α* (7 folds) following Pb intoxication (Fig. 5). This was not, however, associated with any significant changes in the mRNA of VDR in renal tissues. Furthermore, similar observations were also detected in the gene expression of all candidate molecules by the testicular tissues following Pb intoxication, except for the catalysing enzyme, which showed no significant alteration together with a significant inhibition of *VDBP* gene (Fig. 5). Up-regulation in the mRNA of *Cyp27b1*, *VDR*, *CaSR*, *and IL-10* with simultaneous decreases in the mRNA expression of *IL-4* and *TNF-α* in both renal and testicular tissues of the P-VD group, were significantly different compared with the PC group. The results of all genes are illustrated in Fig. 5.

## Discussion

This study measured the reciprocal interactions between chronic lead intoxication and serum VD concentrations concomitantly with the effects of VD_3_ supplementation on several molecular pathways associated with Pb-induced tissue damage in kidneys and testes. The results showed that animals, chronically exposed to Pb, had significantly higher tissue contents of the heavy metal that coincided with significantly lower concentrations of serum 25-OH VD and Ca^2+^. Moreover, the mRNA and protein expression levels of VD synthesising enzyme, VDR and CaSR were downregulated with simultaneous marked increases in VDBP and Cyp24a1 in the kidney and testis of positive control animals.

Our results are in agreement with the findings of numerous earlier human studies that have reported inverse correlations between blood lead levels and the serum concentrations of both VD^29,30,33^ and Ca^2+^ ^34–36^. Skin exposure to ultraviolet rays is the main source of VD in the prohormone form, which is later transported to the liver to undergo the first hydroxylation step. Pb poisoning is known to provoke hepatotoxicity, and therefore, lead-induced liver damage could intervene with VD biogenesis^14^. Alternatively, the observed reduction in circulatory VD and the abnormal cellular expression of its machinery could also be related to Pb-induced nephrotoxicity^1,14^, since the active form of VD is produced in renal proximal tubular cells^21,22^. Therefore, we suggest that excess Pb deposition is tissues could result in abnormal cellular metabolism of VD by concurrently upregulating Cyp24a1 and inhibiting Cyp27b1 enzymes, especially in the major organs responsible for the production of VD, thus resulting in lower blood levels. However, more studies that involve skin and liver specimens are still required to support our proposals regarding the biological interactions between Pb toxicity and VD metabolising system.

Lead poisoning is a worldwide public health hazard and several studies have indicated the deleterious effects of Pb on kidney and testis^12,17^. Studies in humans have shown that high blood Pb level increases the risk of developing kidney diseases by affecting transportation in proximal tubules and diminishing creatinine clearance^14^. Experimental studies have also shown that prolonged exposure to lead resulted in renal cell injury, interstitial necrosis, tubular dysfunction and fibrosis of renal tissues^7,8^. Similarly, humans chronically exposed to Pb had impaired testicular functions in the form of low sperm count and the majority of sperm had abnormal morphology and were immotile^15,16^. Additionally, animal studies have revealed similar effects following chronic administration of Pb, and the testicular tissue showed interstitial degeneration and lower numbers of viable Sertoli, Leydig and germ cells together with significantly lower serum testosterone^37–40^.

The present study correlates with the abovementioned findings, since chronic ingestion of Pb induced the deposition of the toxic element in the kidney and testis, resulting in significant injuries of both organs and showed similar histopathological characteristics reported by earlier studies^7,8,37–40^. Our results also showed a significant increase in the lipid peroxidation marker, MDA, and significant decreases in anti-oxidant markers, GSH, GPx and CAT, in the tissues of interest. Additionally, the PC group had significantly higher levels of the pro-inflammatory cytokines, TNF-α and IL-4, as well as lower level of the anti-inflammatory cytokine, IL-10, in serum and tissues. These findings provide further support to the notion that excess accumulation of Pb in renal and testicular tissues induces damages by increasing cellular ROS, oxidative stress and pro-inflammatory responses^6–8^.

In general, the interrelations between oxidative stress and inflammation are complex since one of them could alternatively be a cause and the other a consequence^41^. Additionally, the currently available data on Pb-induced tissue pathologies suggests that the metal upregulates several pro-inflammatory molecules together with elevations in oxidative stress markers^6–8^. In this context, a variety of human and experimental reports have demonstrated that Pb induces lipid peroxidation and accumulations of ROS in affected tissues, including kidney and testis^5–7^. Pb also promotes several pro-inflammatory cytokines, including TNF-α and IL-4, in serum and tissues^6,42^. Furthermore, the heavy metal have been shown to inhibit the expression of IL-10, a potent anti-inflammatory cytokine, in tissues and serum following chronic toxicity^6,43^. On the other hand, VD is currently regarded as a nutraceutical agent characterised by anti-oxidative and anti-inflammatory actions in addition to its well-established roles in skeletal and calcium homeostasis^22,24^.

VD metabolising and signalling molecules have all been identified in renal tissues as well as male and female reproductive organs from different species^21,44^. The endogenous VD endocrine system has been shown to regulate vital functions in each organ including suppression of inflammation and oxidative stress^44,45^. In this regard, experimental studies have demonstrated that VD simultaneously promoted several anti-oxidant enzymes (e.g. Gpx1, and Prdx1) and downregulated a panel of pro-inflammatory cytokines (e.g. IL-1β, TNF-α) in a rat model of diabetic nephropathy^46^. Consistently, others have also reported that chronic kidney disease induced by indoxyl sulfate in rats resulted in marked oxidative stress and inflammation through the up-regulation of VD catabolising enzyme^47^. Similar results have also been observed in testicular tissues following treatment of diabetic rats with VD^45,48^. Hence, we speculate that the observed pathological alterations in cellular VD system following Pb poisoning could be an additional mechanism by which the heavy metal promotes oxidative stress, inflammation and subsequently damages kidney and testis tissues^47^. In support with our suggestion above, VD_3_ in our study resulted in significant alleviations in renal and testicular tissue injuries observed in animals exposed to Pb-only. VD_3_ also restored and/or preserved the expression of its cellular endogenous molecules compared with positive controls, suggesting a positive feedback regulation, a common phenomenon previously reported by many studies following treatment with VD^49–51^.

Remarkably, none of the earlier studies measured the effects of VD supplementation on the adverse events associated with Pb poisoning. Our findings demonstrated that VD_3_ significantly diminished Pb-induced tissue damage in the kidney and testis. Tissue restoration/preservation was associated with significant increases in anti-oxidative markers, upregulation of the anti-inflammatory cytokine, IL-10, and a marked decline in the levels of IL-4 and TNF-α. Similar observations have been previously reported for VD on oxidative stress markers and the expression of IL-10, IL-4 and TNF-α in chronic renal diseases and testicular dysfunction^44,45^. Hence, the present findings advocate that VD, through its anti-inflammatory and anti-oxidative properties, could negate the toxic effects of excess Pb on renal and testicular cells. However, the hormone had no effects on the tissue Pb concentration, since tissue specimens from the PC and P-VD groups displayed comparable levels of Pb in kidney and testicular specimens. Additionally, the applied VD dose in our study did not provoke hypercalcemia as shown by the biochemistry results. We, therefore, propose that future studies should include Ca^2+^ supplementation with or without higher doses of VD_3_, in order to measure and compare the potential effects of both agents on Pb precipitation in tissues, since the interconnections between VD and Pb could be calcium-dependent^36^. Furthermore, sperm count and morphology should also be included in future studies to affirm full restoration of testicular functions and fertility preservation.

In conclusion, this study is the first to display molecular mutual interactions between vitamin D and Pb toxicity. Additionally, this report demonstrated for the first time that VD_3_ has potential protective effects against Pb-induced renal and testicular injury through anti-inflammatory and antioxidant pathways. Nevertheless, more studies using calcium supplementation, alongside with exogenous VD_3_, are compulsory to explore the protective and therapeutic values of the VD endocrine system against toxicity induced by heavy metals.

## Materials and Methods

### Drugs and chemicals

Lead acetate trihydrate, Pb(CH3CO2)2·3H2O, purity 99.99% was obtained from Sigma-Aldrich Co. (MO, USA) and vitamin D_3_ intramuscular ampoules (100,000 IU/mL) were from Memphis Co. for Pharm. & Chem. Ind. (Cairo, Egypt).

### Study Design and treatment protocols

All the experiments were approved by the Committee for the Care and Use of Laboratory Animals at Umm Al-Qura University and were in accordance with the EU Directive 2010/63/EU for animal experiments.

A total of 32 male Wister rats of 12 weeks of age and 220–250 gm of body weight each were housed in clean and sterile polyvinyl cages (4 rats/cage), maintained on a standard laboratory pellet diet and water ad libitum; and kept in a temperature-controlled air-conditioned at 22–24 °C and 12 h dark/light cycle. The animals were allocated randomly and equally into four groups (8 rats/group): NC group, PC group included animals that only received lead acetate, N-VD group consisted of normal rats injected with VD_3_, and the P-VD group simultaneously received lead acetate and VD_3_. Lead acetate was dissolved in drinking water (1,000 mg/L) and was given for 4 weeks to induce chronic toxicity as previously described^52,53^. VD_3_ was diluted in sterile saline and then given every other day for a total duration of 4 weeks (1,000 IU/Kg; 3 days/week) according to the previously published protocols by Abdelghany et al^24^. and Farhangi *et al*.^54^. However, VD_3_ was administrated intramuscularly in the present study to prevent any potential effect on the absorption rate of lead and/or VD_3_ if both were administrated orally.

### Types of Samples

The animals were anaesthetised using diethyl ether (Fisher Scientific UK Ltd, Loughborough, UK) and 3 ml of blood were obtained from each animal in a plain tube from the middle canthus of the eye just before euthanasia. All blood samples were centrifuged and the serum was stored in −20 °C. The right kidney and testis were collected from each animal, cut in halves and a part from each organ was processed by conventional methods and finally embedded in paraffin. Another piece (0.5 gm) from the remaining half of each organ was digested with 6:1 wet acid ultrapure concentrated nitric acid: Perchloric acid using a Microwave Digestion System. The digested samples were then diluted with ultrapure deionised water and stored at −4 °C till processed to measure the concentrations of Pb.

The remaining left kidney and testis were cut in halves and one piece from each organ was immediately processed for protein extraction using 3 ml of RIPA lysis buffer containing protease inhibitors (Santa-Cruz Biotechnology Inc., CA, USA) and electrical homogeniser. Following centrifugation, small aliquots (0.5 ml) of the resultant supernatant from each sample was placed in Eppendorf tube and the concentrations of total proteins were measured on Qubit Fluorometer (Thermo Fisher Scientific, CA, USA). All extracted renal and testicular total protein samples were then diluted with normal sterile saline for a final concentration of 500 µg/ml and stored in −20 °C.

The residual renal and testicular tissues were immersed in RNA*Later* (Thermo Fisher Scientific) and stored in −80 °C. The extraction of total RNA was done using the Paris kit (Thermo Fisher Scientific) according to the manufacturer’s instructions. The quality of RNA was assessed on a BioSpec-nano machine (Shimadzu Corporation, Tokyo, Japan) and typically had an A260/A280 ratio of 1.7 to 1.9. The quantities of Total RNA samples were measured on Qubit Fluorometer and then aliquoted in 20 ng/µl and stored at −80 °C.

### Lead tissue concentrations

The concentrations of renal and testicular Pb were measured by atomic absorption spectrophotometry (Perkin Elmer AAnalyst 800, MA, USA) with hollow cathode lamp of Pb at wavelength of 283.3 nm as previously described^42^.

### Serum Biochemical Parameters Assay

The serum levels of renal function markers (creatinine and urea), 25-OH vitamin D and calcium were measured using Cobas e411 (Roche Diagnostics International Ltd, Switzerland) according to the manufacturer’s protocols.

### Enzyme linked Immunosorbent Assay (ELISA)

Serum TNF-α, IL-4 and IL-10 were measured using specific rat ELISA kits (R&D systems, MN, USA). All samples were processed in duplicate on a fully automated ELISA system (Human Diagnostics, Germany) according the manufacturer’s guidelines. As reported by the manufacturer, the lowest detection ranges of TNF-α, IL-4 and IL-10 were 3.1 pg/mL, ≤3 pg/mL and 3 pg/mL, respectively. The levels of renal and testicular tissue antioxidant markers GSH, SOD, CAT, GPx and the lipid peroxidation marker, MDA, were also measured by ELISA (Cayman Chemical Co., MI, USA).

### Histology Studies

Sections of 5 μm thickness from each tissue block were stained with Mason’s trichrome (Abcam, MA, USA) to assess fibrosis in the organs of interest. Two expert histopathologists and who were blind to the source group evaluated and scored renal and testicular fibrosis in all sections on an EVOS XL Core microscopy (Thermo Fisher Scientific) using 10 random non-overlapping fields from each slide at ×400 magnification. Additionally, quantitative measurement of collagen deposition (fibrosis index %) was done using ImageJ software (https://imagej.nih.gov/ij/) as previously described^24^ (Supplementary Fig. 1).

### TUNEL Assay

Cell DNA damage and cell apoptosis/necrosis were assessed in the collected tissue specimens using the Click-iT™ TUNEL Alexa Fluor™ 488 Imaging Assay (Thermo Fisher Scientific) and by following the manufacturer’s protocol. Stained slides were examined on an EVOS FL microscopy (Thermo Fisher Scientific) at ×400 magnifications. DNA damage was indicated by the emission of green fluorescence dye and the apoptotic/necrotic cells were counted using the cell counter tool provided with the microscope software (Supplementary Fig. 2). Apoptosis/necrosis index was measured by calculating the percentage of damaged cells in 15 random non-overlapping fields from each tissue section.

### Immunohistochemistry

The primary polyclonal IgG antibodies (Santa-Cruz Biotechnology Inc.) against VDR, CaSR, and VDBP were raised in rabbit, while those against Cyp27b1, Cyp24a1, IL-10 and TNF-α were raised in goat. An avidin-biotin horseradish peroxidase technique using the Elite Vectastain Rabbit or Goat ABC kits (Vector Laboratories Inc., CA, USA) was applied to localise the target proteins by following the manufacturer’s guidelines. The concentrations were 1:200 for all primary antibodies. Negative control slides containing a section from each tissue block under investigation were treated identically to all other slides, but the primary antibodies were replaced with their corresponding primary normal goat or rabbit IgG antibodies (Santa-Cruz Biotechnology Inc.) to control for non-specific staining.

The sections were observed on an EVOS XL Core microscope at ×400 magnification to evaluate the immunostain by two blinded expert observers. The images for each individual protein of interest were captured from 15 random non-overlapping fields from each tissue section. Captured images were later processed for digital image analysis with ImageJ software. Quantification of immunostain colour intensity together with the percentage of area of distribution for each molecule were identified and measured by the Immunohistochemistry (IHC) Image Analysis Toolbox in the software (supplementary Fig. 3) followed by reciprocal immunostain calculation as previously described^55,56^.

### Quantitative RT-PCR

A high capacity RNA-to-cDNA Reverse Transcription Kit (Thermo Fisher Scientific) was used for the synthesis of cDNA from 200 ng of total RNA and by following the manufacturer’s protocol. PCR was executed in triplicate wells on ABI® 7500 system using power SYBR Green master mix (Thermo Fisher Scientific). Each PCR well included 10 µl SYBR Green, 7 µl DNase/RNase free water, 1 µl of each primer (5 pmol; supplementary table 1) and 1 µl cDNA (25 ng) and 40 cycles (95 °C/15 s and 60 °C/1 min) of amplification were performed as previously described^24^. Negative controls included one minus-reverse transcription control from the previous RT step and another minus-template PCR, in which nuclease free water was used as a template. The 2^−∆∆Ct^ method was used to perform relative quantitative gene expression of rat *CYP27B1*, *CYP24A1*, *VDR*, *VDBP*, *CaSR*, *IL4*, *IL10 and TNF-α* genes. Rat *β-actin* gene showed the most consistent results among the three tested reference genes and was used to normalise the Ct values of the genes of interest. The results are expressed as fold-change compared with the NC group.

### Statistical Analysis

SPSS version 16 was used for statistical analysis. All data were assessed for normality and homogeneity by the Kolmogorov and Smirnoff test and Levene test, respectively. One way ANOVA followed by the LSD post hoc test were used to compare between the study groups. P value < 0.05 was considered significant.

### Availability of materials and data

All data generated or analysed during this study are included in this published article (and its Supplementary Information files).

## Electronic supplementary material

Supplementary tables and figures
