# Supplementary material for: Vitamin D alleviates lead induced renal and testicular injuries by immunomodulatory and antioxidant mechanisms in rats
Source: Sci Rep. 2018 Mar 19;8:4853. doi: 10.1038/s41598-018-23258-w (PMC5859277; doi:10.1038/s41598-018-23258-w)
Supplement: Supplementary file 1 — Supplementary tables and figures [file 41598_2018_23258_MOESM1_ESM.pdf]

Vitamin D alleviates lead induced renal and testicular injuries by immunomodulatory and antioxidant mechanisms in rats

Mohammad A. BaSalamah[1], Abdelghany Hassan Abdelghany[2,3], Mohamed El-Boshy[3,4], Jawwad Ahmad[3], Shakir Idris[3] and Bassem Refaat[3*]

Supplementary table 1: The sequences of PCR primers used for the detection of rat β-actin, β2 microglobulin, GAPDH, vitamin D synthesising (*CYP27b1*) and catalysing (*CYP24a1*) enzymes, binding protein (*VDBP*), receptor (*VDR*), calcium sensing receptor (*CaSR*), IL-4, TNF-α and IL-10 mRNAs in renal and testicular samples including the corresponding genes accession numbers and amplicon sizes.

Genes	Forward	Reverse	Amplicon size
ACTB (NCBI: NM_031144.3)	5' CGG TCA GGT CAT CAC TAT CG 3'	5' TTC CAT ACC CAG GAA GGA AG 3'	79 bp
B2M (NCBI: NM_012512.2)	5' TGA AGG AGC CCA AAA CCG TC 3'	5' CCG GAT CTG GAG TTA AAC TGG 3'	92 bp
GAPDH (NCBI: NM_017008.4)	5' GCA TCT TCT TGT GCA GTG CC 3'	5' GAG AAG GCA GCC CTG GTA AC 3'	105 bp
CYP27b1 (NCBI: NM_053763.1)	5' CCC CAC CCA TTT GCA TCT CT 3'	5' CCA ACG CCA TTT GTA GCT CG 3'	91 bp
CYP24a1 (NCBI: NM_201635.3)	5' GGG GCA GAT TTC CTC TGT GA 3'	5' ATC CAC ATC AAG CTG TTC GC 3'	122 bp
VDBP (NCBI: NM_012564.3)	5' GTG GTC AGA GTG CCA CAC AA 3'	5' CGC AGC ATT CCC TAA GGG TT 3'	126 bp
VDR (NCBI: NM_017058.1)	5' CAG CTC TGA CCT GTA CAC CA 3'	5' CAG AGT CAC AGA CGG GTC AT 3'	103 bp
CaSR (NCBI: NM_133315.2)	5' ACT TCA GCA TCC AGC TGT TCC 3'	5' TGT AGA GGC TGC CCG AGA T 3'	103 bp
IL4 (NCBI: NM_201270.1)	5' TGT AGA GGT GTC AGC GGT CT 3'	5' TGT TGT GAG CGT GGA CTC AT 3'	66 bp
TNFα (NCBI: NM_012675.3)	5' CAT CCG TTC TCT ACC CAG CC 3'	5' AAT TCT GAG CCC GGA GTT GG 3'	97 bp
IL10 (NCBI: NM_012854.2)	5' CCT CTG GAT ACA GCT GCG AC 3'	5' TGG CCT TGT AGA CAC CTT TGT 3'	118 bp

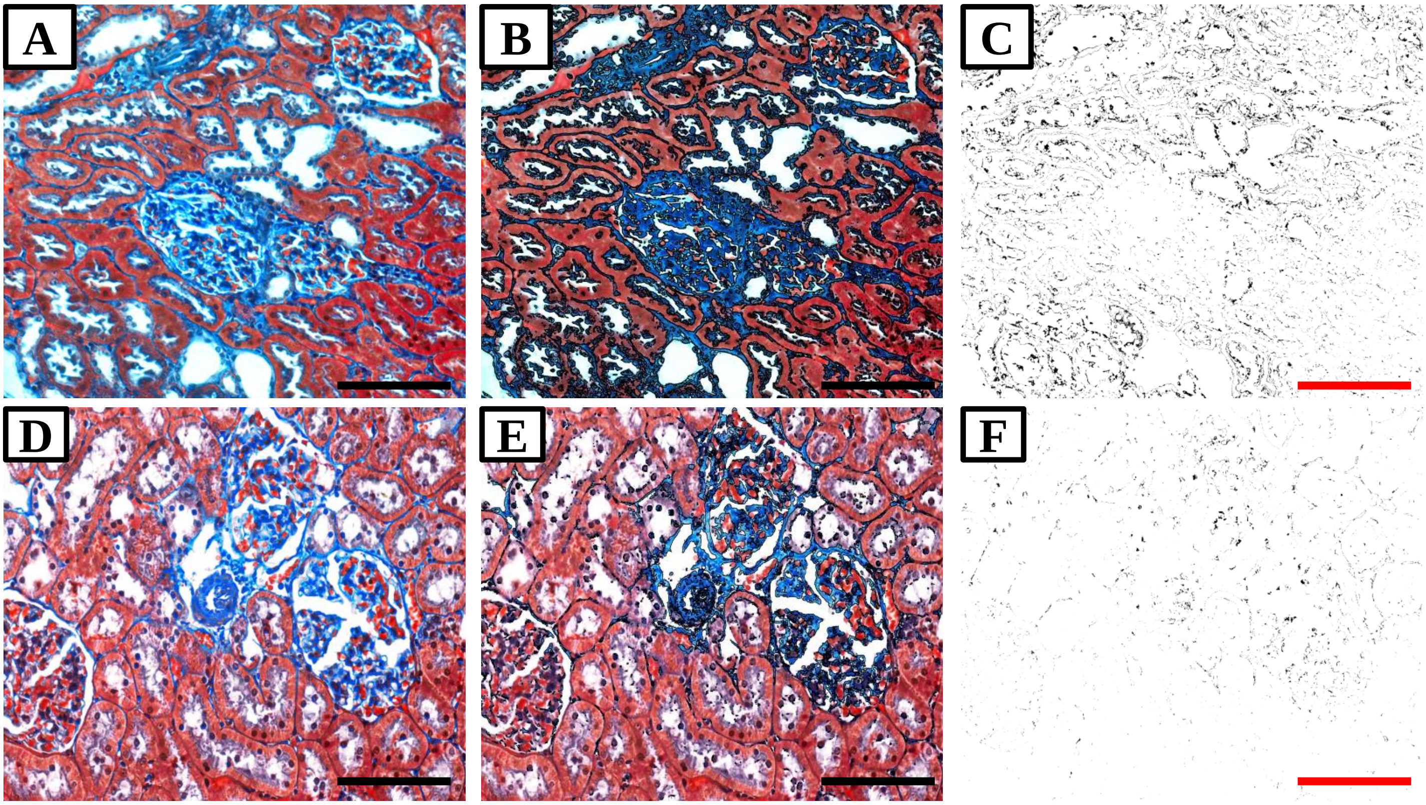

Supplementary Figure 1: Steps of processing the study digital images with ImageJ software for calculating the fibrosis index (% of collagen deposition), which was identified by blue staining, following Masson's trichrome stain of renal sections from PC (upper row) and P-VD treated (bottom row) groups. The identification and selection of the areas of interest (left column) were done with the guidance of an expert histopathologist. The images were then processed by hue/saturation/brightness for colour threshold adjustment using 'black' as the threshold colour to digitally mark and select an area of interest by the software (middle column). This was followed by clearing outside the defined areas of interest to ensure the precision of the identification and selection processes by the software (Right column). All measurements were calculated following calibration with digital photos of corresponding microscopic scale slides captured at the designated magnifications. (40× objective, scale bar = 8 µm).

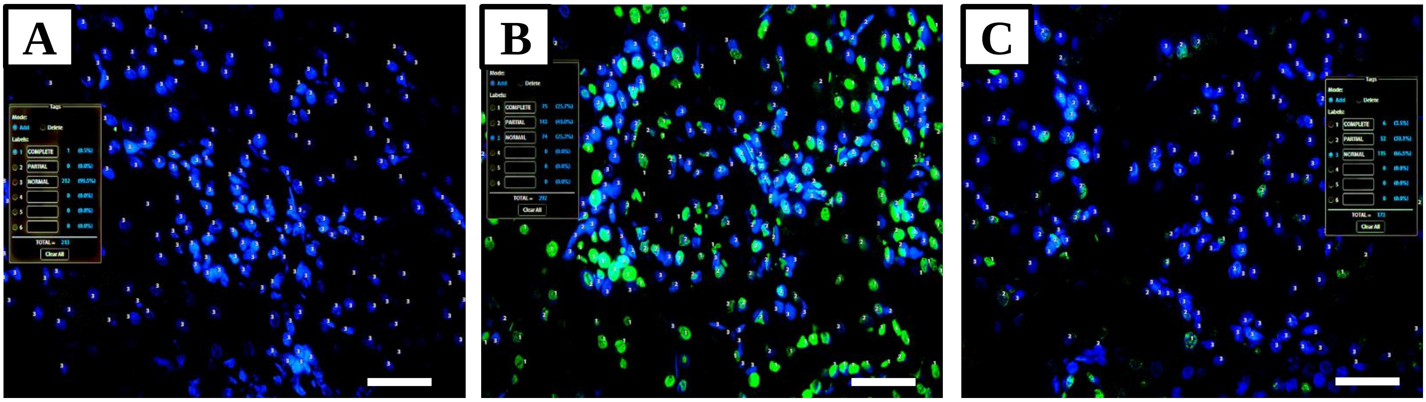

Supplementary Figure 2: Applied method for the calculation of the percentage of apoptotic bodies (green) by the Cell Counter tool provided with the software of EVOS FL microscopy (Thermo Fisher Scientific) in kidney tissue sections from (A) NC, (B) PC and (C) P-VD groups. (40× objective, scale bar = 10 µm).

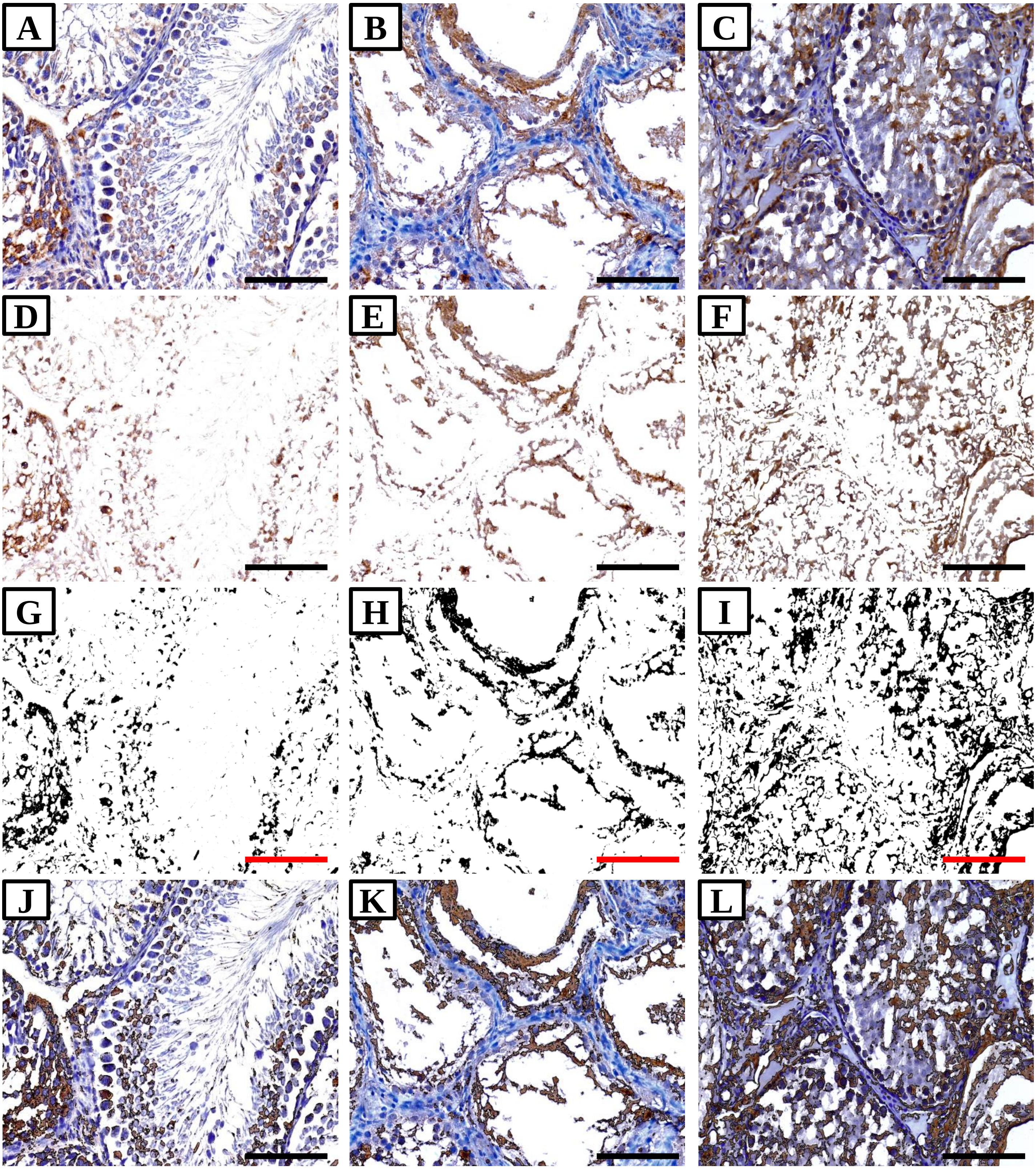

Supplementary Figure 3: Steps of processing the study digital images with ImageJ software for calculating the immunohistochemistry scores of Cyp24a1 enzyme in testicular tissues from NC (left column), PC (middle column) and P-VD (right column) groups. The identification and selection of the areas of interest (2nd row from top) were done by the Immunohistochemistry (IHC) Image Analysis Toolbox plugin. The images were then processed using hue/saturation/brightness for colour threshold adjustment using 'black' as the threshold colour to digitally mark and select an area of interest by the software (3rd row from top). This was followed by merging the selected areas with their corresponding coloured original images to ensure that only the areas of interest were precisely defined and selected by the software (bottom row). The surface area and reciprocal colour intensity were measured on each image to calculate the final immunohistochemistry scores as described in the methodology section. (40× objective, scale bar = 8 µm).
